# 3D-printed GelMA/CaSiO_3_ composite hydrogel scaffold for vascularized adipose tissue restoration

**DOI:** 10.1093/rb/rbad049

**Published:** 2023-05-08

**Authors:** Jupei Zhang, Zhen Zeng, Yanxin Chen, Li Deng, Yanxin Zhang, Yumei Que, Yiren Jiao, Jiang Chang, Zhihong Dong, Chen Yang

**Affiliations:** School of Mechanical Engineering, Chengdu University, Chengdu 610106, China; Joint Centre of Translational Medicine, The First Affiliated Hospital of Wenzhou Medical University, Wenzhou 325000, China; Wenzhou Institute, University of Chinese Academy of Sciences, Wenzhou 325000, China; Joint Centre of Translational Medicine, The First Affiliated Hospital of Wenzhou Medical University, Wenzhou 325000, China; Wenzhou Institute, University of Chinese Academy of Sciences, Wenzhou 325000, China; College of Materials Science and Opto-electronic Technology, University of Chinese Academy of Sciences, Beijing 100049, China; Joint Centre of Translational Medicine, The First Affiliated Hospital of Wenzhou Medical University, Wenzhou 325000, China; Wenzhou Institute, University of Chinese Academy of Sciences, Wenzhou 325000, China; Joint Centre of Translational Medicine, The First Affiliated Hospital of Wenzhou Medical University, Wenzhou 325000, China; Wenzhou Institute, University of Chinese Academy of Sciences, Wenzhou 325000, China; Joint Centre of Translational Medicine, The First Affiliated Hospital of Wenzhou Medical University, Wenzhou 325000, China; Wenzhou Institute, University of Chinese Academy of Sciences, Wenzhou 325000, China; Joint Centre of Translational Medicine, The First Affiliated Hospital of Wenzhou Medical University, Wenzhou 325000, China; Wenzhou Institute, University of Chinese Academy of Sciences, Wenzhou 325000, China; Joint Centre of Translational Medicine, The First Affiliated Hospital of Wenzhou Medical University, Wenzhou 325000, China; Wenzhou Institute, University of Chinese Academy of Sciences, Wenzhou 325000, China; Joint Centre of Translational Medicine, The First Affiliated Hospital of Wenzhou Medical University, Wenzhou 325000, China; Wenzhou Institute, University of Chinese Academy of Sciences, Wenzhou 325000, China; Shanghai Institute of Ceramics, Chinese Academy of Sciences, Shanghai 200050, China; School of Mechanical Engineering, Chengdu University, Chengdu 610106, China; Joint Centre of Translational Medicine, The First Affiliated Hospital of Wenzhou Medical University, Wenzhou 325000, China; Wenzhou Institute, University of Chinese Academy of Sciences, Wenzhou 325000, China

**Keywords:** 3D-printing, bioceramic, composite scaffold, adipogenesis, angiogenesis

## Abstract

The increased number of mastectomies, combined with rising patient expectations for cosmetic and psychosocial outcomes, has necessitated the use of adipose tissue restoration techniques. However, the therapeutic effect of current clinical strategies is not satisfying due to the high demand of personalized customization and the timely vascularization in the process of adipose regeneration. Here, a composite hydrogel scaffold was prepared by three-dimensional (3D) printing technology, applying gelatin methacrylate anhydride (GelMA) as printing ink and calcium silicate (CS) bioceramic as an active ingredient for breast adipose tissue regeneration. The *in vitro* experiments showed that the composite hydrogel scaffolds could not only be customized with controllable architectures, but also significantly stimulated both 3T3-L1 preadipocytes and human umbilical vein endothelial cells in multiple cell behaviors, including cell adhesion, proliferation, migration and differentiation. Moreover, the composite scaffold promoted vascularized adipose tissue restoration under the skin of nude mice *in vivo*. These findings suggest that 3D-printed GelMA/CS composite scaffolds might be a good candidate for adipose tissue engineering.

## Graphical Abstract

**Figure:**
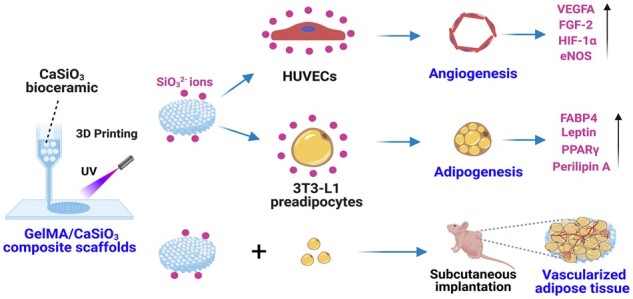

## Introduction

The restoration of adipose tissue is required in many clinical scenarios, such as mastectomies following breast cancer, which usually result in the absence of breast tissue and cause severe psychological and physiological burdens to patients. Although current strategies, including lipofilling [1, 2], autologous-tissue flap transplantation [3] and silicone prostheses implantation, have been widely practiced for breast reconstruction [4], they have numerous drawbacks and limitations, such as uncontrollable resorption of fats, source limitation of autologous-tissue flap and capsule contracture of silicone prosthesis [5]. All these disadvantages increase the risk of delayed adipose tissue regeneration and failure of breast repair. Besides, since breast reconstruction is particularly personalized, additional breast plastic surgery is usually required after the treatments of these strategies due to the lack of precise shape control [6]. Therefore, the development of an innovative treatment strategy with high activity for adipose tissue regeneration and precise structure control ability for implanted construct is urgently needed.

Three-dimensional (3D)-printed scaffold-guided adipose tissue engineering is attracting more and more attention as a promising technique to repair injured breasts [7], especially for large-volume defects [8, 9], in which the scaffold is utilized as a ‘bridge’ to facilitate cellular interactions and tissue formation [10, 11]. Compared to conventional scaffolds made of thermoplastic materials (e.g. polycaprolactone), the 3D printing of hydrogels represents an advantageous alternative because of their extracellular matrix-mimicking structural network and good biomimetics of adipose tissue mechanical properties [12]. Among several hydrogels used for 3D-printed scaffolds (alginate [13, 14], hyaluronic acid [15], collagen [16], silk fibroin [17], chitosan [18], etc.), gelatin methacrylate anhydride (GelMA) is a promising candidate in adipose tissue engineering due to its ease of crosslinking by ultraviolet (UV) irradiation, low antigenicity and good biocompatibility [19]. For example, Petra *et al.* proved that both human primary mature adipocytes and adipose-derived stem cells could survive well in the 3D-printed GelMA hydrogels with the phenotype of adipocytes [20]. Nevertheless, the activity of adipose tissue regeneration mediated by this single-component GelMA scaffold is not significant enough for clinical application.

It is well established that the efficiency of adipogenic differentiation and sufficient vascularization are crucial for successful adipose tissue reconstruction [21]. Accordingly, several endogenous angiogenic factors (e.g. basic fibroblast growth factor (bFGF) [22] and insulin-like growth factor 1 (IGF-1) [23]) have been applied to incorporate with 3D-printed scaffold to induce better adipogenesis and angiogenesis [24]. However, their inherent disadvantages such as high cost, difficulty to be accurately released and short half-life *in vivo* limit their applications in clinical practice [25]. To date, scaffolds that can simultaneously meet both the need for personalization of the breast structure and promotion of lipogenesis/vascularization are still scarce [26].

Silicate biomaterials have recently been developed for the repair of various injured tissues, such as skin [27, 28], bone [29], tendon [30], endometrium [31], aorta [32] and heart [33]. The sustained release of the bioactive ions from these materials was proved to make the main contribution to the beneficial effects on tissue regeneration [34, 35]. For example, silicate ions derived from calcium silicate (CS, CaSiO_3_) bioceramic could stimulate pro-angiogenesis of human umbilical vein endothelial cells (HUVECs) *in vitro*, and enhance neovascularization in mouse infarcted myocardium [36]. More interestingly, our previous study demonstrated that CS extract promoted adipogenic differentiation of human bone marrow mesenchymal stem cells (hBMSCs) by stimulating key regulators relative to adipogenesis, and suppressed dedifferentiation of hBMSC-derived adipocytes in a cocultured system with HUVECs by mediating their interactions [37]. All these studies indicate that CS may serve as a bioactive ingredient for the promotion of adipogenesis and angiogenesis simultaneously.

**Figure 1.**
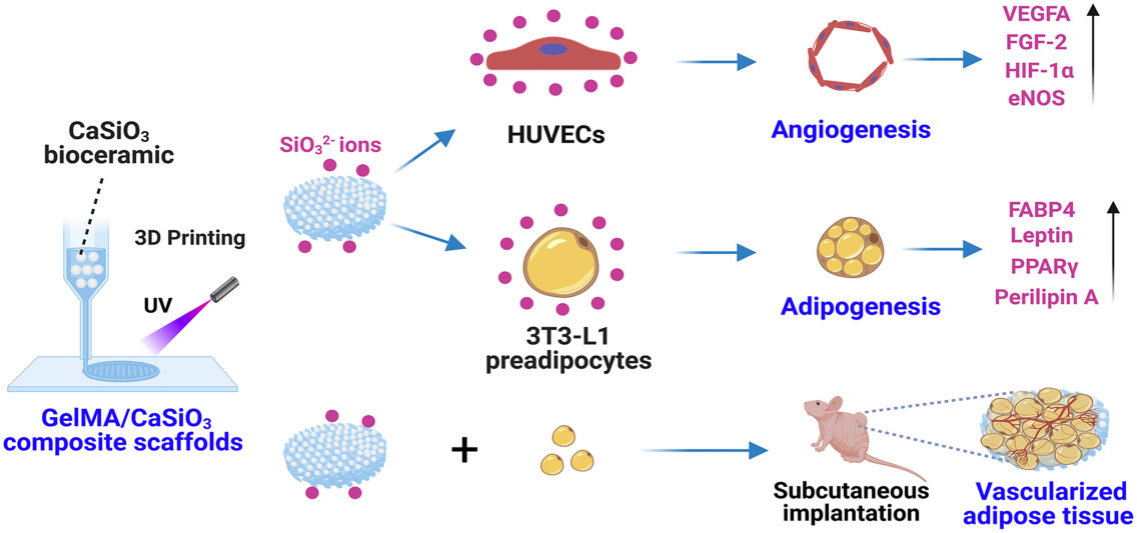
Schematic diagram of the 3D-printed GelMA/CS composite hydrogel scaffolds for adipose tissue regeneration. Figure 1 was created with BioRender.com.

Based on the above considerations, we hypothesize that 3D-printed GelMA/CS composite hydrogel scaffolds might be a good candidate for adipose tissue regeneration (Fig. 1). To verify our hypotheses, in this study, we developed 3D-printed GelMA/CS composite scaffolds with different concentrations and investigated their physicochemical properties, followed by exploring their adipogenic effect on 3T3-L1 preadipocytes and angiogenic effect on HUVECs. Finally, we reconstructed an engineered adipose tissue based on 3D-printed GelMA/CS composite scaffolds in a nude mice subcutaneous implant model, and evaluated the performance in stimulating vascularized adipose tissue regeneration.

## Materials and methods

### Materials

GelMA was purchased from Cure Gel Co., Ltd (Zhejiang, China). CS (CaSiO_3_) was purchased from Kunshan Chinese Technology New Materials Co, Ltd (Jiangsu, China). Cell counting kit-8 (CCK-8) was purchased from Yeasen Biotechnology Co., Ltd (Shanghai, China). Oil Red O was purchased from Aladdin (Shanghai, China). Isobutyl methylxanthine (IBMX), insulin (Human Recombinant), indomethacin and dexamethasone were purchased from Sigma-Aldrich (USA). All primers were purchased from Sangon Biotech (Shanghai, China). Platelet endothelial cell adhesion molecule-1 (CD31) was purchased from Servicebio (Wuhan, China). Peroxisome proliferators-activated receptors (PPARγ) was purchased from Beyotime (Shanghai, China).

### 3D printing of GelMA/CS composite scaffolds

The bio-ink for 3D printed GelMA/CS composite scaffold was first prepared by mixing different weight concentrations (0.2, 0.4, 0.8 wt%) of CaSiO_3_ powder in GelMA solution (1 g GelMA, 10 ml deionized water) containing photoinitiator (0.05 g) away from light. Then, a 3D printer (BioMaker4, SunP Biotech, Beijing, China) was used to produce GelMA/CS composite hydrogel scaffolds by applying light-curing assisted extrusion technology with the settings of printing speed of 5 mm/s, extrusion speed of 1 mm^3^/s and time curing of 15 s for each layer under 365 nm UV light. The scaffolds of 20 mm × 20 mm × 2 mm following a crossed lay-dawn pattern (45°) were prepared, cut into cylinders (diameter of 5 mm and 8 mm) and freeze-dried for future use. As a control, pure GelMA scaffolds were also 3D printed using the same protocol.

### Characterization of 3D-printed GelMA/CS composite scaffolds

The morphology and element mapping of the scaffolds was observed using a scanning electron microscope (SEM, HITACHI SU8010, Japan). The compressive mechanical property of scaffolds was measured using an Instron machine (Instron 5944, USA) by exerting pressure on the composite scaffold with a displacement speed of 3 mm/min. Ten consecutive load–unload cycles were applied to characterize the fatigue resistance of the gel. For the *in vitro* degradation analysis, scaffolds were immersed in Tris–HCL solution (pH = 7.4) with a weight-to-volume ratio of 1 g/20 ml and shaken in an incubator shaker with the speed of 120 rpm/min at 37°C for 21 days. At setting the timepoints (0, 1, 3, 7, 14 and 21 days), the scaffolds were freeze-dried and weighted, respectively. Meanwhile, the Tris–HCL solution was collected to determine their released silicate ions using an inductively coupled plasma mass spectrometry (ICP-MS, Agilent 7850, USA).

The porosity of scaffolds was determined using the ethanol displacement method [38]. Briefly, a vial filled with ethanol was weighed as M0 and the scaffold was weighed as M1, respectively. Then, the scaffold was put into the vial slowly until all the pores were infiltrated with ethanol. The vial filled with ethanol and scaffold was weighed as M2. Finally, the scaffold was taken out and the vial containing residual ethanol was weighed as M3. The percentage porosity (φp) was calculated according to the following equation:


φp=(M2–M3–M1)/(M0–M3)×100%

### *In vitro* cell studies

#### Cell culture

3T3-L1 preadipocytes were purchased from the Chinese National Immortalized Cell Bank and cultured in high glucose Dulbecco’s modified eagle medium (DMEM) supplemented with 10% fetal bovine serum (FBS) and 1% penicillin/streptomycin solution (P/S) in a humid atmosphere of 5% CO_2_ at 37°C. HUVECs were purchased from EK-Bioscience (Shanghai, China) and cultured in high glucose DMEM supplemented with 10% FBS and 1% P/S in a humid atmosphere of 5% CO_2_ at 37°C.

#### Cell proliferation assay

CCK-8 assay kit was used to determine the cell proliferation at 1, 3 and 5 days, respectively. Briefly, 3T3-L1 preadipocytes or HUVECs were seeded on different scaffolds with a cell density of 2 × 10^3^ cells/well in 96 well plates, respectively. Then, the cell culture media were replaced with CCK-8 solution and incubated for 1 h. The absorbance of the supernate at 450 nm was recorded using a microplate reader (Epoch2, Bio-Tec Instruments, USA).

#### Cytocompatibility of the GelMA scaffold

The cytocompatibility of the GelMA scaffold was assessed by using both the CCK-8 assay kit and the Live/Dead Cell kit (Yeasen Biotechnology, Shanghai, China). Briefly, 3T3-L1 preadipocytes or HUVECs were seeded on 96 well plates with a cell density of 2 × 10^3^ cells/well in the presence/absence of GelMA scaffold. After culturing for 1 day, cells were evaluated by the CCK-8 assay kit or the Live/Dead Cell kit following the manufacturer’s instrument. According to the manufacturer’s protocol, calcein‐AM (green fluorescence) represents viable cells and propidium iodide (PI, red fluorescence) represents dead cells.

#### Cell migration

The scratch assay was applied to evaluate the migration performance of either 3T3-L1 preadipocytes or HUVECs co-cultured with scaffolds. Briefly, cells were seeded into the 6-well plate at a concentration of 5 × 10^5^ cells/well and cultured for 24 h to form a monolayer. Then, a straight line was uniformly drawn on the bottom of the plate by a 200-μl pipette tip. Subsequently, scaffolds were placed into the medium assisted by the transwell chamber. After being co-cultured with different scaffolds for 24 h, the cells were fixed with 4% paraformaldehyde and stained with crystal violet for 10 min. Photographs were taken by the microscope (OLYMPUS CKX53, Japan) and the cell migration rate was quantified using Image J software.

#### Adipogenic induction of 3T3-L1 preadipocytes on scaffolds

3T3-L1 preadipocytes were seeded on different scaffolds and first cultured in the normal medium for 2 days. Then, two types (A and B) of adipogenic medium were utilized for further culture: Type A adipogenic medium contained 0.5 × 10^−3^M IBMX, 10 µg/ml insulin, 100 × 10^−6^M indomethacin and 1 × 10^−6^ M dexamethasone, while type B adipogenic medium contained 10 µg/ml insulin. Briefly, type A adipogenic medium (for 3 days) and type B adipogenic medium (for 1 day) were alternately used for two cycles, and then changed to type B adipogenic medium for another 4 days. The total time for adipogenic induction was 14 days.

#### Cell morphology

The morphologies of 3T3-L1 preadipocytes or HUVECs on scaffolds before and after adipogenic induction were visualized by SEM imaging. Briefly, the cell-scaffold constructs were fixed with 2.5% glutaraldehyde for 3 h and dehydrated by graded ethanol (30%, 50%, 70%, 80%, 90% and 100%). The obtained samples were sputter-coated with gold and observed by SEM (HITACHI SU8010, Japan). The morphologies of HUVECs culture on scaffolds for 1 day were also evaluated using the same procedure.

#### Oil Red O staining

After adipogenic induction, 3T3-L1 cells on scaffolds were stained with Oil Red O solution for 20 min and rinsed with phosphate-buffered saline (PBS) three times. The stained cell-scaffold constructs and cells on the culture plate surrounding the scaffolds were visualized by the microscope of OLYMPUS SZ61RT (Japan) and OLYMPUS CKX53 (Japan), respectively. For quantitative analysis, cells were lysed in 100% isopropanol for 15 min and the absorbance was measured by a microplate reader (Epoch2, Bio-Tec Instruments, USA) at 492 nm.

#### Tube formation of HUVECs

Matrigel matrix was first thawed at 4°C overnight, put into a pre-cooled 48-well plate (120 μl/well), and incubated at 37°C for 1 h. Then, HUVECs (4 × 10^4^/well) were seeded on matrigels and co-cultured with different scaffolds for 6 h. Finally, formed tubes were observed by a microscope (OLYMPUS CKX53, Japan) and analyzed using the Image J software.

#### Real-time quantitative PCR (qRT-PCR)

The total RNA of 3T3-L1 cells cultured on scaffolds for 14 days (adipogenic induction) and HUVECs cultured on scaffolds for 3 days were extracted by the Cell Total RNA Kit, respectively. Then the RNA was reverse transcribed into cDNA using the SYBR Premix Ex Taq (Takara, China). The mRNA levels of fatty acid-binding protein 4 (FABP4), Leptin, peroxisome proliferators-activated receptors (PPARγ), Perilipin A in 3T3-L1 cells and vascular endothelial growth factor (VEGFA), hypoxia inducible factor-1 (HIF-1α), basic fibroblast growth factor (FGF-2) and endothelial nitric oxide synthases (eNOS) in HUVECs were determined by qRT-PCR. The relative gene expression was normalized to the housekeeping gene glyceraldehyde-3-phosphate dehydrogenase (GAPDH). The primer sequences used in this study were listed in Supplementary Table S1.

### *In vivo* adipose tissue restoration

#### Nude mice subcutaneous implantation

Six-week-old female nude mice were purchased from the Experimental Animal Center of Zhejiang Province. The animal experimental protocol of this study was approved by the Animal Research and Ethics Committee of Wenzhou Institute of University of Chinese Academy of Sciences (Approval Issue No. WIUCAS22021801). The implantation experimental groups were divided into four groups: pure GelMA scaffold (GelMA), GelMA/CS (0.4 wt%) composite scaffold (0.4% CS), pure GelMA scaffold pre-cultured with 3T3-L1 cells in lipogenic differentiation medium for 14 days (GelMA+cell) and GelMA/CS (0.4 wt%) composite scaffold pre-cultured with 3T3-L1 cells in lipogenic differentiation medium for 14 days (0.4% CS+cell). Scaffolds were implanted on both sides of the mice’s back between the skin and myofascia. After feeding for 3 weeks, the mice were sacrificed and the implanted scaffolds were taken out for the appearance analysis of the formed adipose tissue and blood vessels. Image J software was applied for the evaluation of the volume of adipose tissue/scaffold construct. Throughout the experiment, all mice were housed in the specific pathogen-free (SPF) animal facility of the Experimental Animal Center of Wenzhou Research Institute, University of Chinese Academy of Sciences.

#### Histological and immunohistochemical staining analysis

First, the taken-out samples were fixed in 4% paraformaldehyde, embedded in optimal cutting temperature (OCT SAKURA 4583, UK) and stored at −80°C. Subsequently, samples were sliced into 10 µm thin slices, then stained with Oil Red O solution (Aladdin, Shanghai), and counterstained with hematoxylin. For immunostaining staining, PPARγ as the primary antibody was used to analyze the adipose tissue and primary antibody CD31 was used to analyze blood vessels. Briefly, the sliced samples were rinsed with PBS for 3 min, three times for each one. Then sliced samples were treated with methanol containing 3% H_2_O_2_ for 10 min to block endogenous peroxidase activity, and placed in an oven at 37°C for antigen repair for 1–2 h, then rinsed again with PBS. After being blocked with BSA (5%, 5 g BSA dissolved in 100 ml PBS) for 30 min at 37°C, the sections were incubated at 4°C overnight with primary antibody against PPARγ (1:200) or CD31 (1:400). Then, the fluorescent secondary antibodies by 1:200 dilution was applied for 1 h at room temperature and the images were photographed by the microscope (ZEISS Axio Vert.A1, Germany). Quantitative analysis was performed by Image-Pro Plus software.

### Statistical analysis

All data are expressed as mean ± standard error of mean (SEM). Statistical significance analysis was performed using a one-way analysis of variance (ANOVA). The sample numbers were *n* ≥ 3 and *P* < 0.05 was considered as statistically significant difference, where *P < 0.05, **P < 0.01 and ***P < 0.001. All statistical analyses were performed using GraphPad Prism software (GraphPad Software, USA).

## Results

### Characterization of the 3D-printed GelMA/CS composite scaffolds

**Figure 2.**
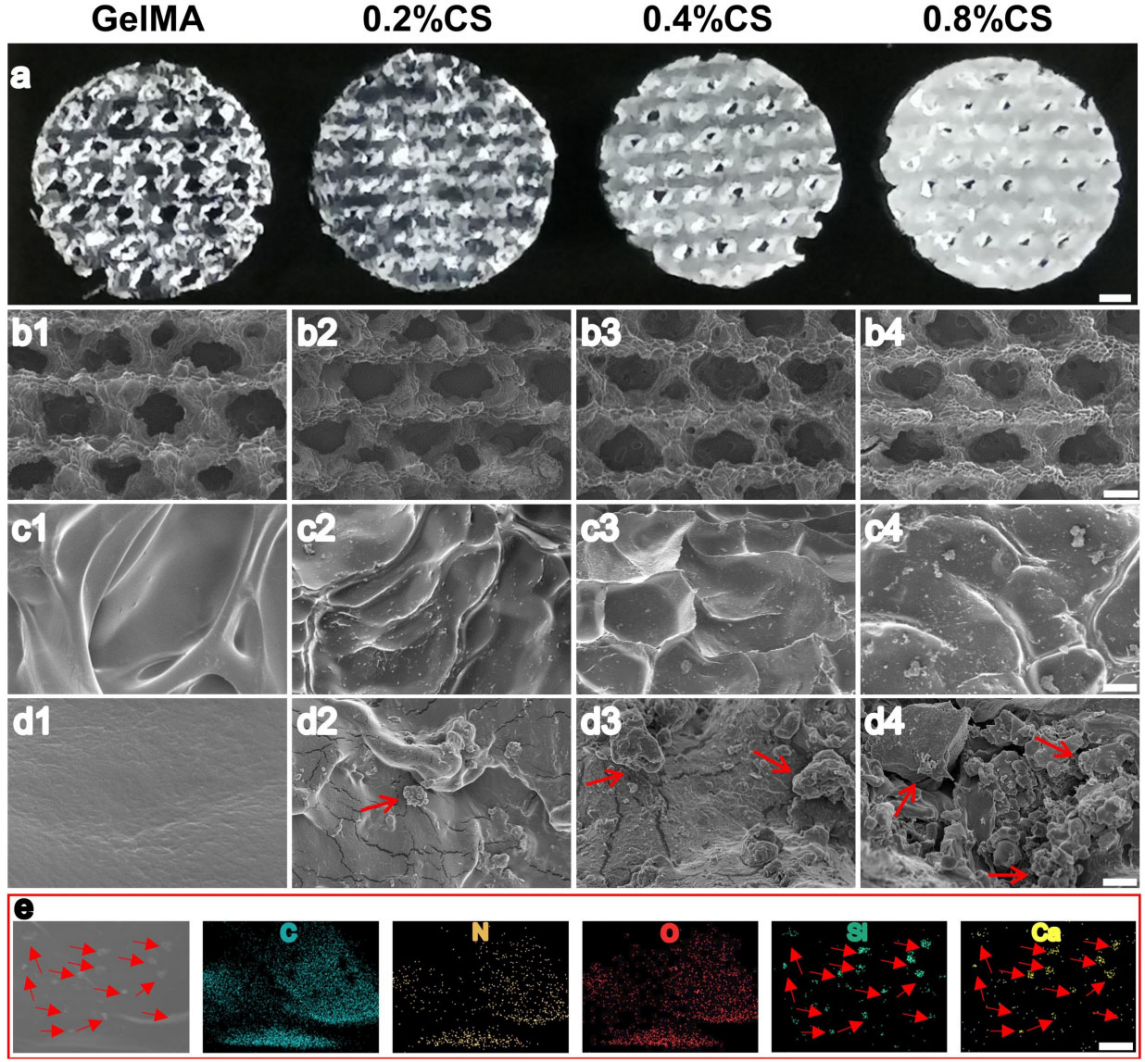
Characterization of 3D printed GelMA/CS composite scaffolds. (**a**) Overview of 3D-printed GelMA/CS composite scaffolds with various concentrations of CS powders. (**b1–b4**) SEM images of macropore structures of the 3D-printed scaffolds. (**c1–c4**) SEM images of surface morphologies of the 3D-printed scaffolds. (**d1–d4**) SEM images of cross-view of the 3D-printed scaffolds. (**e**) EDS mapping of 3D-printed 0.4% CS GelMA composite scaffolds. Red arrow indicates CS powder. Scale bar: 1000 µm (a), 500 µm (b1–b4), 30 µm (c1–c4), 3 µm (d1–d4), 15 µm (e).

GelMA/CS composite scaffolds with various concentrations of CS powders (0.2, 0.4 and 0.8 wt%, denoted as 0.2% CS, 0.4% CS and 0.8% CS) were fabricated through an UV-assisted extrusion-based 3D printing technique. The optimal images of the 3D-printed scaffolds were shown in Fig. 2a. As a control, pure GelMA scaffolds were transparent, while the transparency of GelMA/CS composite scaffolds decreased along with the addition of CS powders. No obvious transparent difference was observed in each scaffold, indicating the homogeneous distribution of CS powders. Then, the surface morphologies of the scaffolds were evaluated by SEM images (Fig. 2b–d). As shown in Fig. 2b1–b4, the macroporous structures of all scaffolds were uniform with a diameter of about 1 mm. However, pure GelMA scaffolds possessed smooth surfaces while the surfaces of GelMA/CS composite scaffolds were apparently rougher due to the incorporated CS powders (Fig. 2c1–c4). As expected, more embedded CS particles (red arrow, cross-section) were observed inside the composite scaffolds along with the increase of CS from the cross-section view (Fig. 2d1–d4). The element mapping analysis of the composite scaffold further confirmed the embedded CS powders (pointed by the red arrow) were homogeneously distributed in the hydrogel matrix (Fig. 2e). Moreover, to explore the customization ability of GelMA/CS composite, a pair of breast-liked scaffolds with a diameter > 8 mm and height > 6 mm were designed and well-fabricated by 3D printing, which demonstrated that 3D-printed GelMA/CS composite scaffolds may be able to match adipose defect with irregular shape (Supplementary Fig. S1). All these results confirmed the successful fabrication of 3D-printed GelMA/CS composite scaffolds.

**Figure 3.**
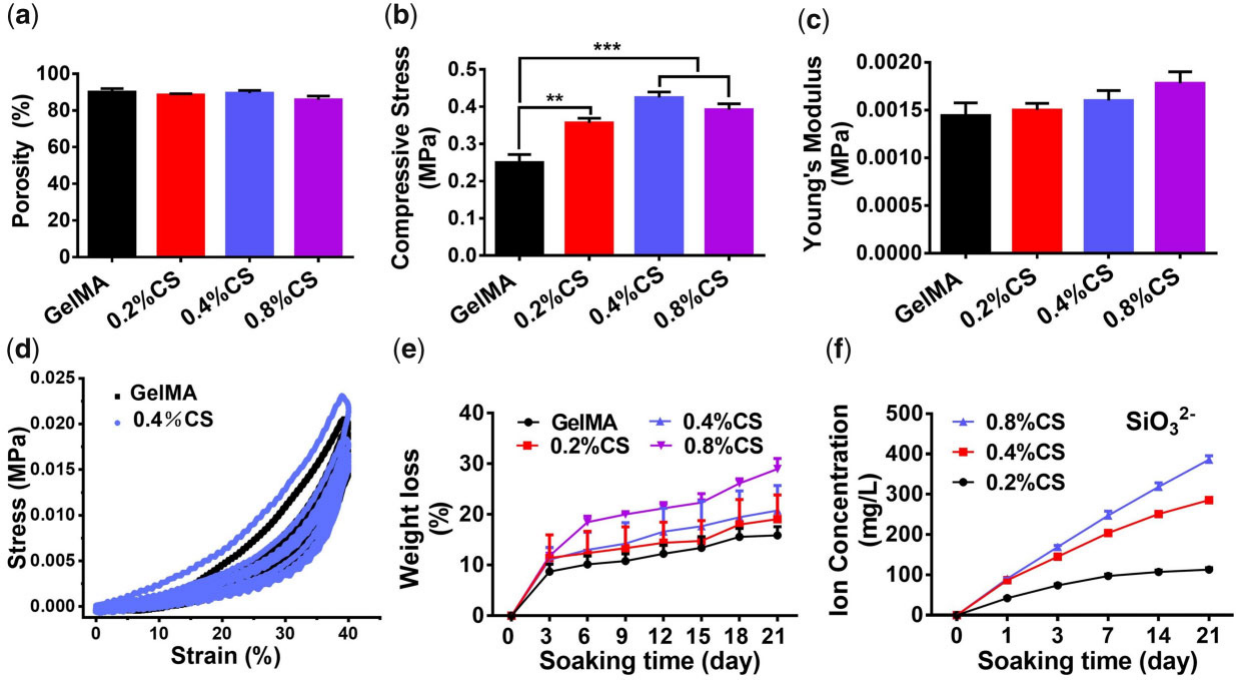
Physiochemical properties of the 3D-printed scaffolds. (**a**) The porosity of scaffolds (*n* = 4). (**b**) The compressive strength of scaffolds (*n* = 5). (**c**) The Young’s modulus of scaffolds (*n* = 5). (**d**) Stress–strain curve of the cyclic compressive test. (**e**) The *in vitro* degradation of scaffolds (*n* = 3). (**f**) The accumulated concentration of released ${\text{SiO}}_{3}^{2-}$ ions (*n* = 3). **P < 0.01, ***P < 0.001.

The physicochemical properties of the 3D-printed scaffolds were further explored and presented in Fig. 3. Although there was no significant difference of the porosity (∼90%) between different scaffolds (Fig. 3a), the compressive strengths of the composited scaffolds (0.33 ± 0.02 ∼ 0.39 ± 0.02 MPa) were significantly higher than the pure GelMA scaffolds (0.2504 ± 0.02091 MPa), especially for the group of 0.4% CS, which displayed the highest compressive strengths (Fig. 3b). The compressive Young’s moduli were also elevated in the composited scaffolds as compared to the pure GelMA scaffolds, and 0.8% CS composite scaffolds possessed the highest Young’s modulus (Fig. 3c). Moreover, as the cyclic compressive test showed, both GelMA and 0.4% CS composite scaffolds possessed high mechanical stability as no obvious changes were shown after 10 cycles in both the pure GelMA scaffolds and 0.4% CS composite scaffolds (Fig. 3d), indicating the good stability of the fabricated scaffolds. The *in vitro* degradation assay exhibited that all scaffolds could be degraded with time. After 21 day’s soaking in Tris–HCl solution, the degradation rate of the scaffolds reached 15.90 ± 1.69% (GelMA), 19.09 ± 4.77% (0.2% CS), 20.77 ± 4.94% (0.4% CS) and 28.99 ± 2.06% (0.8% CS), respectively, showing the uptrend along with the content of CS (Fig. 3e). In addition, the ions release profile of different composite scaffolds was evaluated. There was no surprise as both ${\text{SiO}}_{3}^{2-}$ and Ca^2+^ ions were sustainedly released from all composite scaffolds, and the composite scaffolds with higher concentrations of CS had more ions released (Fig. 3f and Supplementary Fig. S2). It is worth mentioning that the release curves of ${\text{SiO}}_{3}^{2-}$ ions and Ca^2+^ ions showed inconsistent in shape, which may be ascribed to the adsorption of Ca^2+^ by the carboxyl group in GelMA.

### 3D-printed GelMA/CS composite scaffolds promote the proliferation, migration and adipogenic differentiation of 3T3-L1 cells

Before evaluating the adipogenic and angiogenic effects of the composite scaffolds, the good cytocompatibility of the synthetic GelMA was first confirmed by coculturing 3T3-L1 preadipocytes or HUVECs with GelMA scaffolds. Both the CCK-8 assay and the Live/Dead assay (Supplementary Fig. S4) demonstrated the absence of cytotoxic effects of the GelMA scaffold (Supplementary Fig. S4).

**Figure 4.**
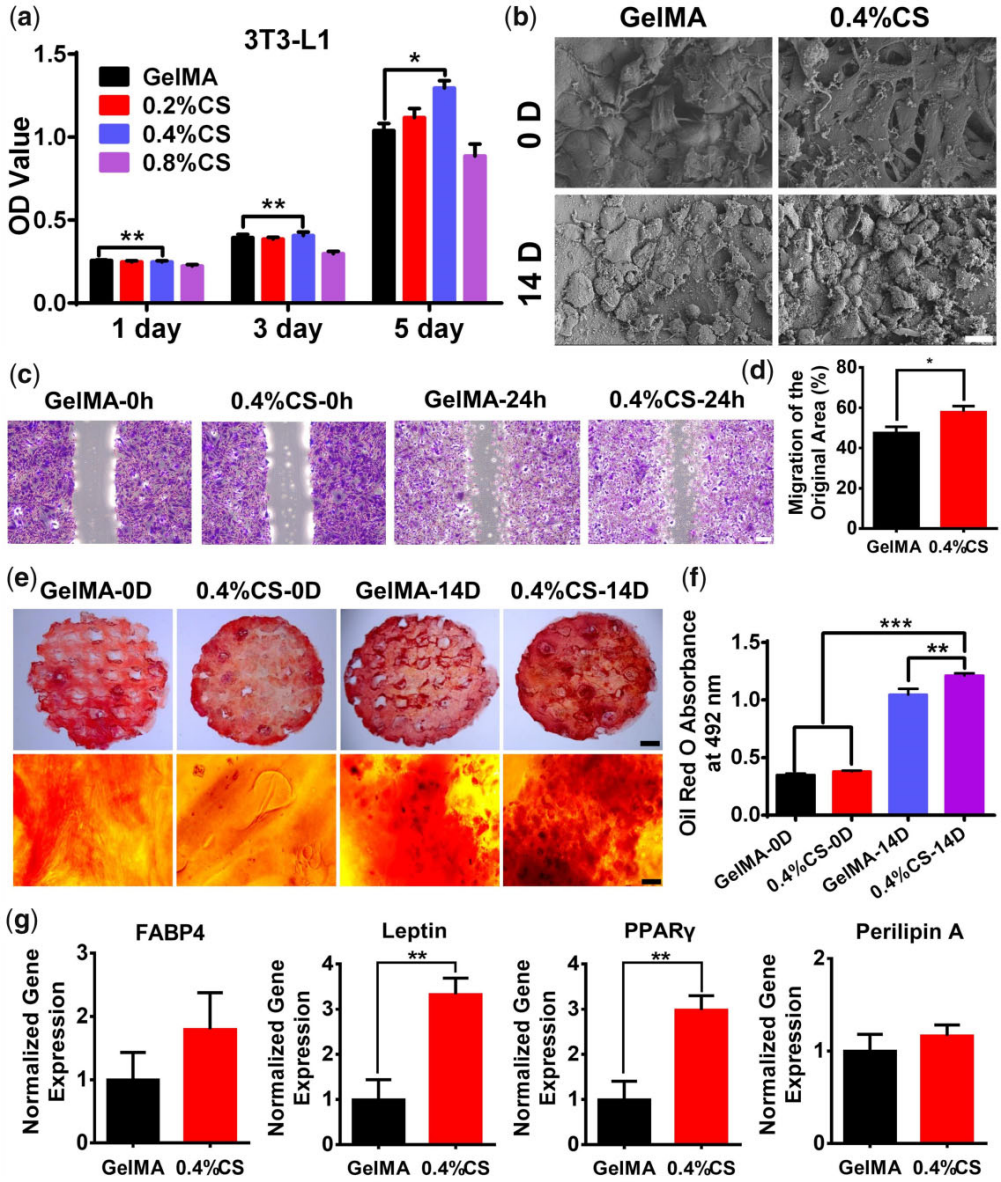
The effect of 3D-printed scaffolds on 3T3-L1 cells. (**a**) The proliferation of 3T3-L1 cells on different scaffolds for 1, 3 and 5 days, respectively (*n* = 5). (**b**) Representative SEM images of 3T3-L1 cells cultured on different scaffolds before and after adipogenic induction for 14 days (*n* = 3). (**c**) Representative images of cell migration with the treatment of pure GelMA and 0.4% CS composite scaffolds for 24 h using the scratch assay. (**d**) Quantitative analysis of cell migration rate (*n* = 6). (**e**) Representative Oil Red O staining micrographs of different scaffolds before and after adipogenic induction for 14 days. (**f**) Quantitative analysis of Oil Red O staining (*n* = 5). (**g**) Adipogenic gene (FABP4, leptin, PPARγ and perilipinA) expression of 3T3-L1 cells cultured on 3D-printed GelMA and 4% CS composite scaffolds after adipogenic induction for 14 days (*n* = 6). Scale bar: 15 μm (b), 200 μm (c), 1 mm (e, upper row), 30 μm (e, lower row). *P < 0.05, **P < 0.01, ***P < 0.001.

The *in vitro* adipogenic performance of the scaffolds was evaluated using 3T3-L1 preadipocytes. The cell proliferation ability of the scaffolds was first estimated and the result was shown in Fig. 4a. Cells proliferated on all scaffolds from day 1 to day 5, while 0.4% CS composite scaffolds displayed the best proliferation compared with other groups at all timepoints, and a significant difference was shown between the pure GelMA scaffolds and 0.4% CS composite scaffolds. Therefore, we chose 0.4% CS composite scaffold as a representative composite scaffold for further study. The SEM images exhibited that 3T3-L1 cells attached well on both pure GelMA scaffolds and 0.4% CS composite scaffolds, and differentiated into adipocytes after incubation in the adipogenic induction media for 14 days as the cell shape changed from fibrous to round (Fig. 4b). Then, the cell migration performance with the treatment of scaffolds was evaluated using the scratching method and the result revealed that 0.4% CS composite scaffolds significantly promoted a higher cell migration rate than GelMA scaffolds (Fig. 4c and d). The adipogenic differentiation of 3T3-L1 cells on different scaffolds was further confirmed by the Oil Red O staining (Fig. 4e and f). There was no significant difference between pure GelMA scaffolds and 0.4% CS composite scaffolds before adipogenic induction. However, more lipid accumulation was observed on 0.4% CS composite scaffolds than pure GelMA scaffolds with an increment of about 16% after adipogenic induction for 14 days, indicating the better adipogenic ability of composite scaffolds. Furthermore, the effect of scaffolds on adipogenic gene expression was examined by qRT-PCR and the results were shown in Fig. 4g. About 0.4% CS composite scaffolds upregulated the various gene expressions including FABP4, leptin, PPARγ and perilipin A as compared to pure GelMA scaffolds, and significant differences were displayed in leptin and PPARγ. All these outcomes suggested the beneficial effect of 0.4% CS composite scaffolds on adipogenesis.

### 3D-printed GelMA/CS composite scaffolds promote the proliferation, migration, tube formation and pro-angiogenesis of HUVECs

**Figure 5.**
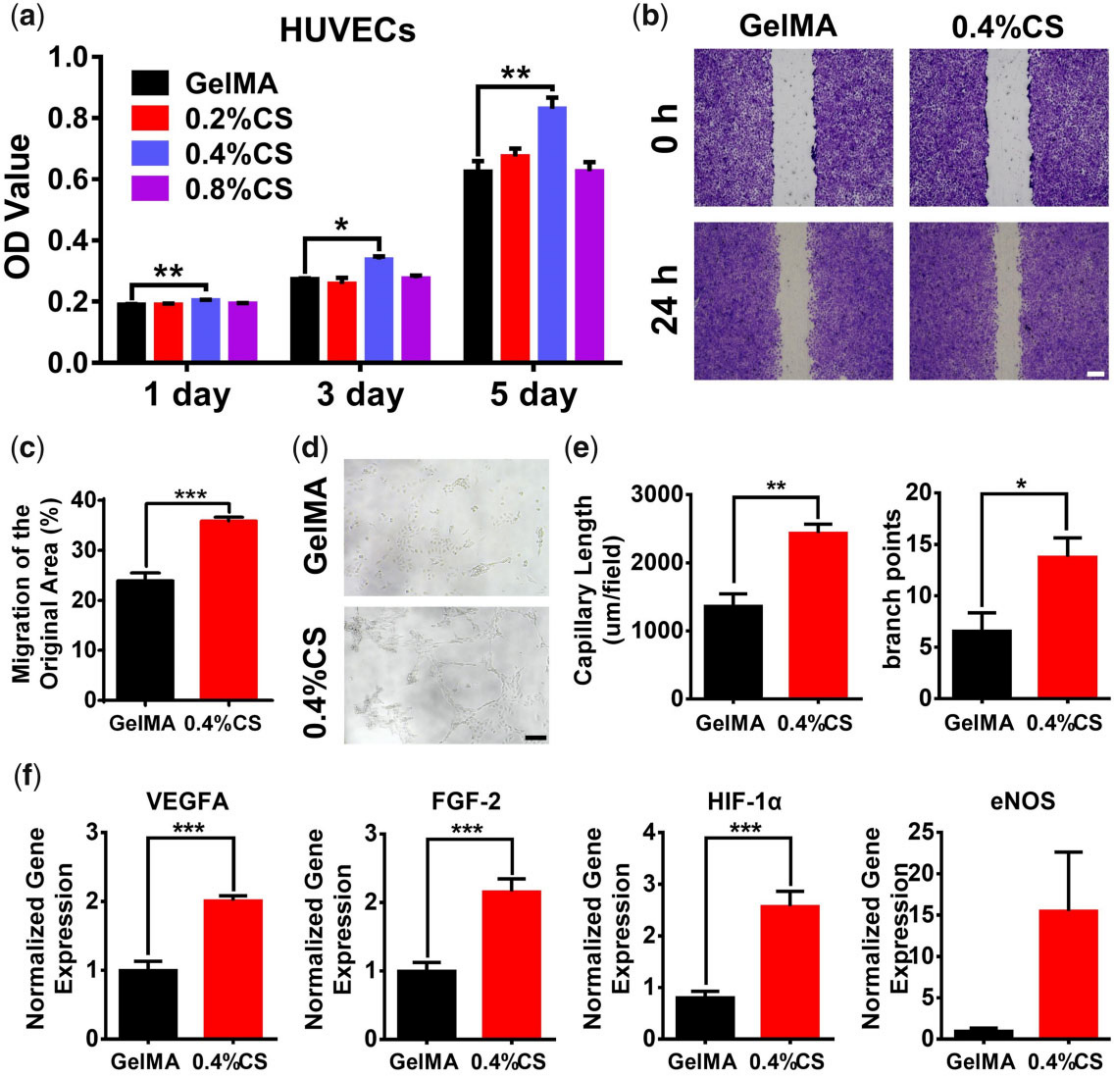
The effect of 3D-printed scaffolds on HUVECs. (**a**) Cell proliferation on different 3D-printed scaffolds for 1, 3 and 5 days, respectively (*n* = 5). (**b**) Representative images of cell migration with the treatment of pure GelMA and 0.4% CS composite scaffolds for 24 h using the scratch assay. (**c**) Quantitative analysis of cell migration rate (*n* = 8). (**d**) Representative images and (**e**) quantitative analysis of the *in vitro* tube formation assay of HUVECs after being treated with pure GelMA and 0.4% CS composite scaffolds for 6 h (*n* = 5). (**f**) Angiogenic genes (VEGFA, HIF-1α, eNOS and FGF-2) expression in HUVECs cultured on pure GelMA and 0.4% CS composite scaffolds for 3 days (*n* = 6). Scale bar: 200 μm (b) and 120 μm (d). *P < 0.05, **P < 0.01, ***P < 0.001.

The *in vitr*o angiogenic performance of the scaffolds was evaluated using HUVECs. The HUVECs proliferation ability of the scaffolds was first investigated, which had a similar tendency to 3T3-L1 cells as 0.4% CS composite scaffolds displayed the best proliferation compared with other groups at day 1, day 3 and day 5 (Fig. 5a). Therefore, 0.4% CS composite scaffolds were chosen for further studies. SEM images revealed that HUVECs were well spread on both GelMA and 0.4% CS composite scaffolds, indicating good cell attachment of all the 3D-printed scaffolds (Supplementary Fig. S3). However, the scratch assay proved that the cell migration rate of HUVECs with the treatment of 0.4% CS composite scaffolds (35.86 ± 0.76%) for 24 h was significantly higher than that cultured with pure GelMA scaffolds (23.91 ± 1.59%) (Fig. 5b and c). The *in vitro* tube formation assays further exhibited that more tubes formed in Matrigel after co-cultured with 0.4% CS composite scaffolds for 6 h as compared to pure GelMA scaffolds (Fig. 5d), which was confirmed by the quantitative data of both the capillary length (≈1.8-fold) and branch points (≈2.1-fold) (Fig. 5e). Moreover, angiogenic genes (VEGFA, FGF-2, HIF-1α and eNOS) expression in HUVECs were detected by qRT-PCR. As shown in Fig. 5f, 0.4% CS composite scaffolds significantly upregulated the gene expression of VEGFA, FGF-2 and HIF-1α in HUVECs after culturing for 3 days in comparison to pure GelMA scaffold. Together, these results suggested the beneficial effect of 0.4% CS composite scaffolds on angiogenesis.

### 3D-printed GelMA/CS composite scaffolds enhanced vascularized adipose tissue regeneration *in vivo*

**Figure 6.**
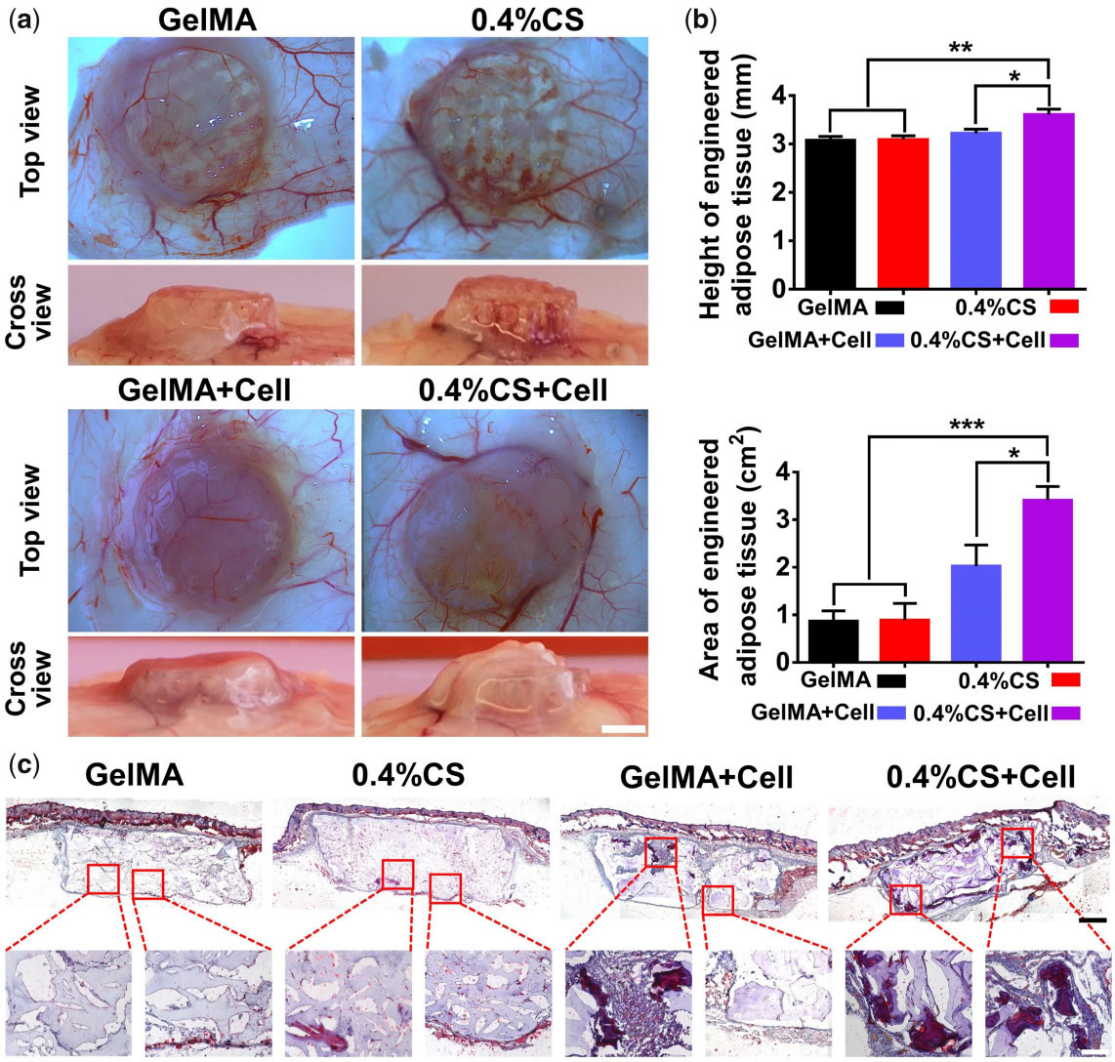
Characterization of the engineered adipose tissues in a nude mice subcutaneous transplantation model by different 3D-printed scaffolds (GelMA, 0.4% CS, GelMA+cell and 0.4% CS+cell) for 3 weeks. (**a**) Representative photos of taken-out engineered adipose tissues. (**b**) Quantitative analysis of the height and area of the engineered adipose tissues (*n* = 6). (**c**) Representative images of Oil Red O staining of the engineered adipose tissues. Scale bar: 2.5 mm (a), 1 mm (c, upper row), 200 μm (c, lower row). *P < 0.05, **P < 0.01, ***P < 0.001.

**Figure 7.**
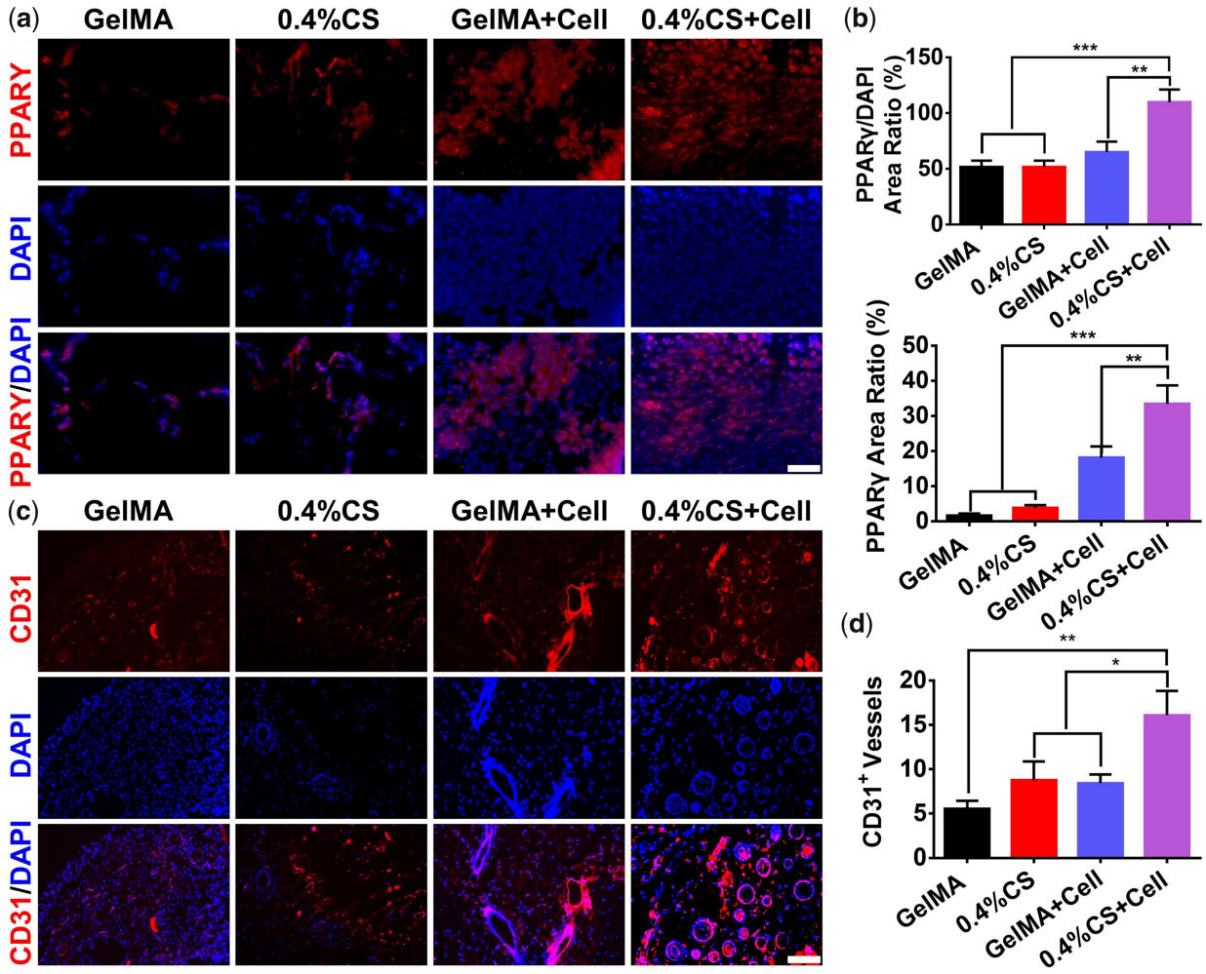
Immunofluorescence staining targeting PPARγ (adipogenic mark) and CD31 (angiogenic mark) in new formed tissues after the treatment with different 3D-printed scaffolds (GelMA, 0.4% CS, GelMA+cell, and 0.4% CS+cell) for 3 weeks. (**a**) Representative immunofluorescence images and (**b**) quantitative analysis of PPARγ in new formed tissues. PPARγ was stained with red fluorescence, and the nucleus was stained with DAPI (*n* = 12). (**c**) Representative immunofluorescence images and (**d**) quantitative analysis of CD31 in new formed tissues. CD31 was stained with red fluorescence, and the nucleus was stained with DAPI (*n* = 9). Scale bar, 40 µm. *P < 0.05, **P < 0.01, ***P < 0.001.

To further investigate the effects of the 3D-printed scaffolds on the regeneration of adipose tissue *in vivo*, pure GelMA and 0.4% CS composite scaffolds pre-cultured with (designated as GelMA+Cell and 0.4% CS+Cell) and without 3T3-L1 cells were subcutaneously implanted in nude mice for 3 weeks, respectively. Figure 6a showed the appearance of engineered adipose tissue, which were constructed by scaffold and newly formed adipose-like tissue. It is clear to see that scaffolds with 3T3-L1 cells (GelMA+Cell and 0.4% CS+Cell) had more tissue formation with visible vessel networks inside as compared with cell-free scaffolds (GelMA and 0.4% CS), while the group of 0.4% CS+Cell promoted the best adipose-like tissue regeneration, which as demonstrated by the quantitative analysis of engineered adipose tissue including the height and the area (Fig. 6b). The Oil Red O staining further confirmed the optical observation as 0.4% CS composite scaffolds remarkably improved the formation of adipose-like tissue in the presence of 3T3-L1 cells (Fig. 6c and Supplementary Fig. S5). Moreover, to evaluate the quality of the newly formed adipose-like tissue, the immunofluorescent staining of PPARγ (adipogenic mark) and CD31 (angiogenic mark) was conducted. As shown in Fig. 7a and b, both newly formed tissues and PPARγ^+^ tissues were observed in cell-containing scaffolds (GelMA+Cell and 0.4% CS+Cell) compared with cell-free scaffolds (GelMA and 0.4% CS), and the highest expression of PPARγ was presented in the group of 0.4% CS+Cell, indicating the promotion effect on adipogenesis by 0.4% CS composite scaffolds. Similarly, more CD31 expression was recorded in cell-containing scaffolds as compared to cell-free scaffolds (GelMA and 0.4% CS) and 0.4% CS+Cell exhibited the best angiogenic promotion effect (Fig. 7c and d). All these results pinned the conclusion that 3D-printed 0.4% CS composite scaffolds hold great potential as grafts for vascularized adipose tissue regeneration.

## Discussion

For patients with a breast reconstruction demand, restoring the appearance of the missing breast as perfectly as possible is the most concerning problem. Commercial breast implantation scaffolds (such as silicone) have shaping characteristics to a certain extent, but they are far from meeting the highly personalized clinical needs of breast reconstruction [39]. To achieve a good breast repair effect at one time, both the macroscopic and internal structures of the scaffolds need to be carefully designed. Specifically, the macroscopic structure (e.g. shape, size and symmetry) of the scaffold needs to meet client’s personalized requirements for ‘natural beauty’, while the internal structure of the scaffold needs to be favorable for adipose tissue regeneration [26]. 3D printing technology is undoubtedly one of the most important techniques in terms of personalized customization and control of scaffold structure, which can not only customize the implanted scaffolds to exactly match the breast defect but also fabricate the scaffolds into well-designed porous structures to achieve better nutrient delivery and cell/tissue growth [40, 41]. Here, we prepared GelMA/CS scaffolds by 3D printing, and demonstrated their potential for personalized customization with well-controlled porous structures. Such 3D-printed GelMA/CS scaffolds were able to load and support the growth of both adipocytes and endothelial cells, showing the capacity for adipose tissue engineering.

Besides architecture, the mechanical property is also crucial for adipose tissue regeneration [42]. From a biomechanical perspective, a successful scaffold should exhibit a low elastic modulus akin to the native tissue, which is beneficial for adipogenesis [43]. In addition, it should be strong enough to permit surgical handling during implantation. This may partially explain the unsuccess of non-scaffold breast reconstruction engineering and the unsatisfied adipose regeneration efficiency of high-strength scaffolds such as polycaprolactone (PCL) due to the mismatched mechanical support in defects [38, 44]. To avoid this issue, this study applied GelMA hydrogel-based scaffold with an average Young’s modulus of 2.15 ± 0.11 kPa, which is close to that of normal human breast dispose of 0.5–25 kPa [45]. The incorporated CS powders further increased both the compressive strength and Young’s modulus of the scaffolds, and the results showed that the 3D GelMA/CS composite scaffold indeed promoted the adhesion and proliferation of 3T3-L1 preadipocytes and HUVECs. Accordingly, our scaffold had mechanical properties similar to those of native tissue and, thus, exhibited better pro-cell growth.

In addition to promoting the growth of adipose-relative cells, stimulating preadipocytes to differentiate into adipocytes and increasing the capillary formation within newly formed adipose tissues are the two key factors for the restoration of adipose tissue [21]. In this regard, incorporating growth factors within implants or utilizing an acellular tissue matrix are the two typical approaches to enhance the bioactivity of adipogenesis and angiogenesis simultaneously. Several growth factors including bFGF, VEGF and IGF-1 have been explored to be added into different 3D grafts for combined angiogenesis and adipogenesis stimulation with optimization focused on concentration and release rate [22–24, 46, 47]. Unfortunately, no commercial product containing growth factors for adipose tissue regeneration is released until now, which reflects the challenge of controlled and targeted growth factor delivery. In contrast, acellular tissue matrix grafts (e.g. AlloDerm^®^), which contain multiple growth factors and cell-favorable 3D structures, have been widely used in various types of breast reconstruction surgery as the assistant of the prosthesis and play a positive role in enhancing adipose regeneration and accelerating prosthesis fusion [48]. However, except for the high cost, the low mechanical strength and rapid degradation rate make them hardly be considered as independent scaffold materials in breast reconstruction, especially for large defects. In this study, we found that CS bioceramic could serve as an active ingredient in 3D-printed GelMA/CS scaffolds for the promotion of adipogenesis by upregulating PPARγ, Leptin, FABP4 and angiogenesis by upregulating VEGFA, FGF-2, HIF-1α, due to the sustained release of silicate ions. Moreover, more vascularized adipose tissue was observed in 3D-printed GelMA/CS composite scaffolds as compared to pure 3D-printed GelMA scaffolds in animal experiment. Considering the low cost, biosafety, stability and diversity of bioceramics, such biocreamic-enhanced composite scaffolds might be easier for clinical transformation compared with growth factors or acellular tissue matrix approaches.

One result of this study is worth mentioning here. The ${\text{SiO}}_{3}^{2-}$ ions and Ca^2+^ ions were all released from CS bioceramic, however, the release curve of ${\text{SiO}}_{3}^{2-}$ ions and Ca^2+^ ions showed inconsistent. The reasons for this result may be 2-fold: on the one hand, this result may be due to errors in ICP-MS measurements; on the other hand, the carboxyl group in GleMA adsorbs part of Ca^2+^, causing its release to be slow at the beginning and gradually increase with time until it gradually becomes the same as ${\text{SiO}}_{3}^{2-}$.

In this study, the traditional cell culture spreading method was used instead of using the latest 3D bioprinting technology. The aim was to ensure the stability of the operation and cell survival, and directly verify the effects of the scaffolds on vascularized adipose tissue regeneration. 3D bioprinting has become an innovative engineering strategy for regenerative medicine, that can build complex biomimetic structures by printing different cells precisely in different locations [49, 50]. However, 3D-bioprinted constructs are typically poorly evaluated post-printing for long-term cell survival, phenotype maintenance and function [51]. Although advances have been made in the development of innovative printing methodologies and new bioinks, key principal engineering paradigms for cell printing applications such as the shear stress field or the crosslinking method remain that have yet to be fully resolved [52]. The results of this study demonstrate the significant effect of CS-containing scaffolds on adipose regeneration, suggesting that this scaffold could be of great value in conjunction with future research on more advanced bioprinting technologies.

## Conclusion

In summary, a novel 3D-printed GleMA/CS composite hydrogel scaffold with capacity of customization and enhanced adipogenic/angiogenic ability was successfully prepared. The obtained scaffolds possessed controllable architecture and enhanced mechanical property. Cell experiments demonstrated that a suitable addition of CS in scaffolds not only promoted the adhesion, proliferation and migration of 3T3-L1 cells and HUVECs, but also enhanced the adipogenesis of 3T3-L1 cells and angiogenesis of HUVECs. The *in vivo* study further confirmed that 3D-printed GleMA/CS composite scaffolds supported the growth of adipocytes and stimulated effectively vascularized adipose restoration. Our study broadens the avenue to engineer customized bioactive materials-based scaffolds for breast reconstruction and adipose tissue regeneration.

## Supplementary Material

rbad049_Supplementary_Data
