# Supplementary material for: 3D-printed GelMA/CaSiO3 composite hydrogel scaffold for vascularized adipose tissue restoration
Source: Regen Biomater. 2023 May 8;10:rbad049. doi: 10.1093/rb/rbad049 (PMC10234763; doi:10.1093/rb/rbad049)
Supplement: rbad049_Supplementary_Data [file rbad049_supplementary_data.zip › Supplementary Materials.docx]

**Table1.** The primer sequences used in this study.

| Target gene | Forward primer sequence  (5′–3′) | Reverse primer sequence  (5′–3′) |
| --- | --- | --- |
| GAPDH | CAG GAG AGT TCC TCG TCC | TTT GCC GTG AGT GGA GTC AT |
| FABP4 | GGT GGT GGA ATG TGT TAT G | ATT GCT TGC TTA TTA GTG GAA |
| Leptin | TCA ACT CCC TGT TTC CAA AT | TCT TCA CGA ATG TCC CAC GA |
| PPARγ | GGA GCC TAA GTT TGA GTT TGC TGT G | TGC AGC AGG TTG TCT TGG ATG |
| Perilipin A | CTT TCT CGA CAC ACC ATG GAA ACC | CCA CGT TAT CCG TAA CAC CCT TCA |
| VEGFA | ATC GAG TAC ATC TTC AAG CCA T | GTG AGG TTT GAT CCG CAT AAT C |
| HIF-1α | ATC CAT GTG ACC ATG AGG AAA T | CTC GGC TAG TTA GGG TAC ACT T |
| FGF-2 | AAA AGG CAA GAT GCA GGA GA | TTT TGC AGC CTT ACC CAA TC |
| eNOS | GAT GTT ACC ATG GCA ACC AAC | GAA AAT GTC TTC GTG GTA GCG |
|  |  |  |


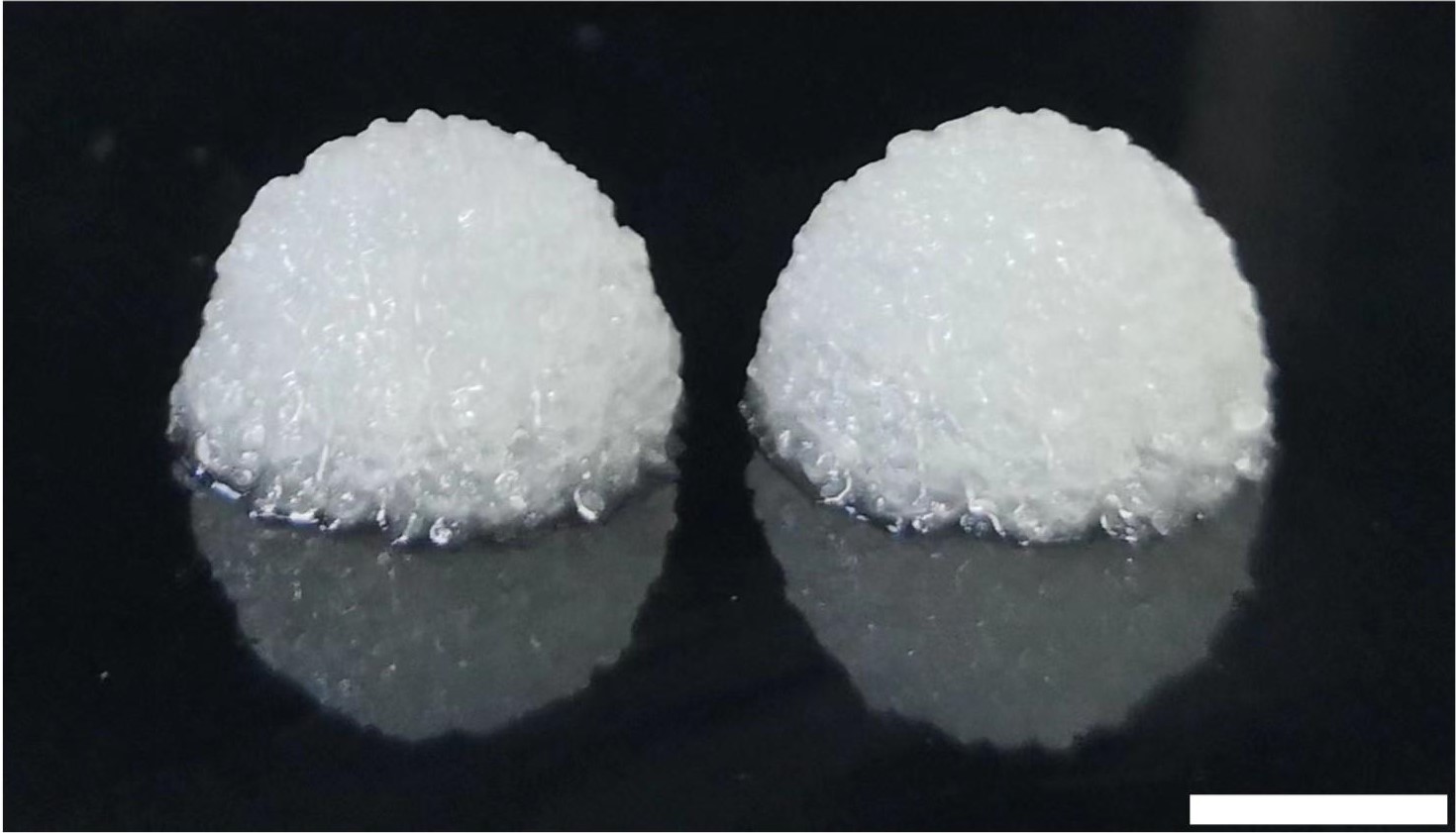


**Figure s1.** Optical images of 3D printed of breast-liked scaffolds. Scale bar: 4mm.


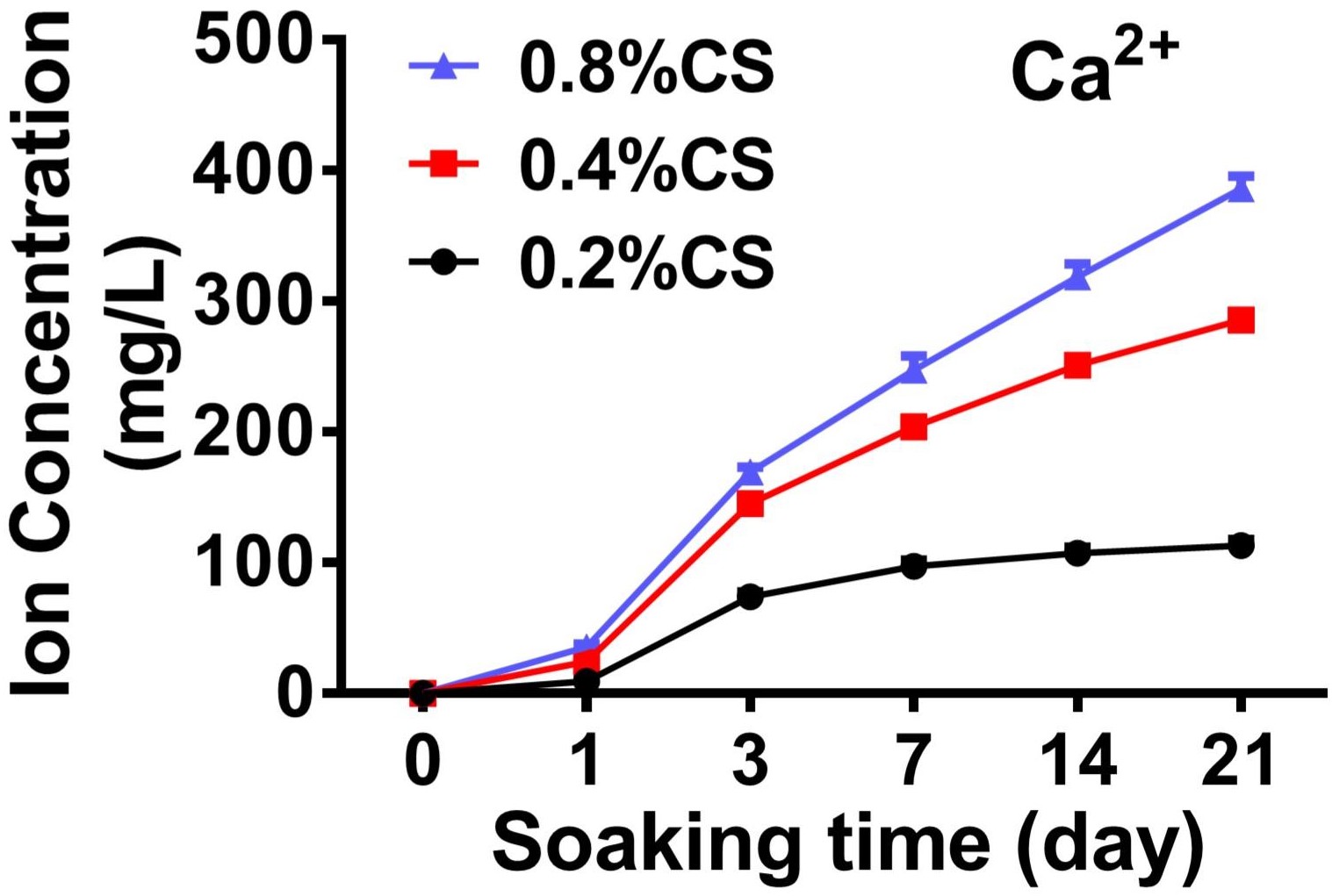


**Figure s2.** The accumulated concentration of released Ca^2+^ ions from different scaffolds (n = 3).

**
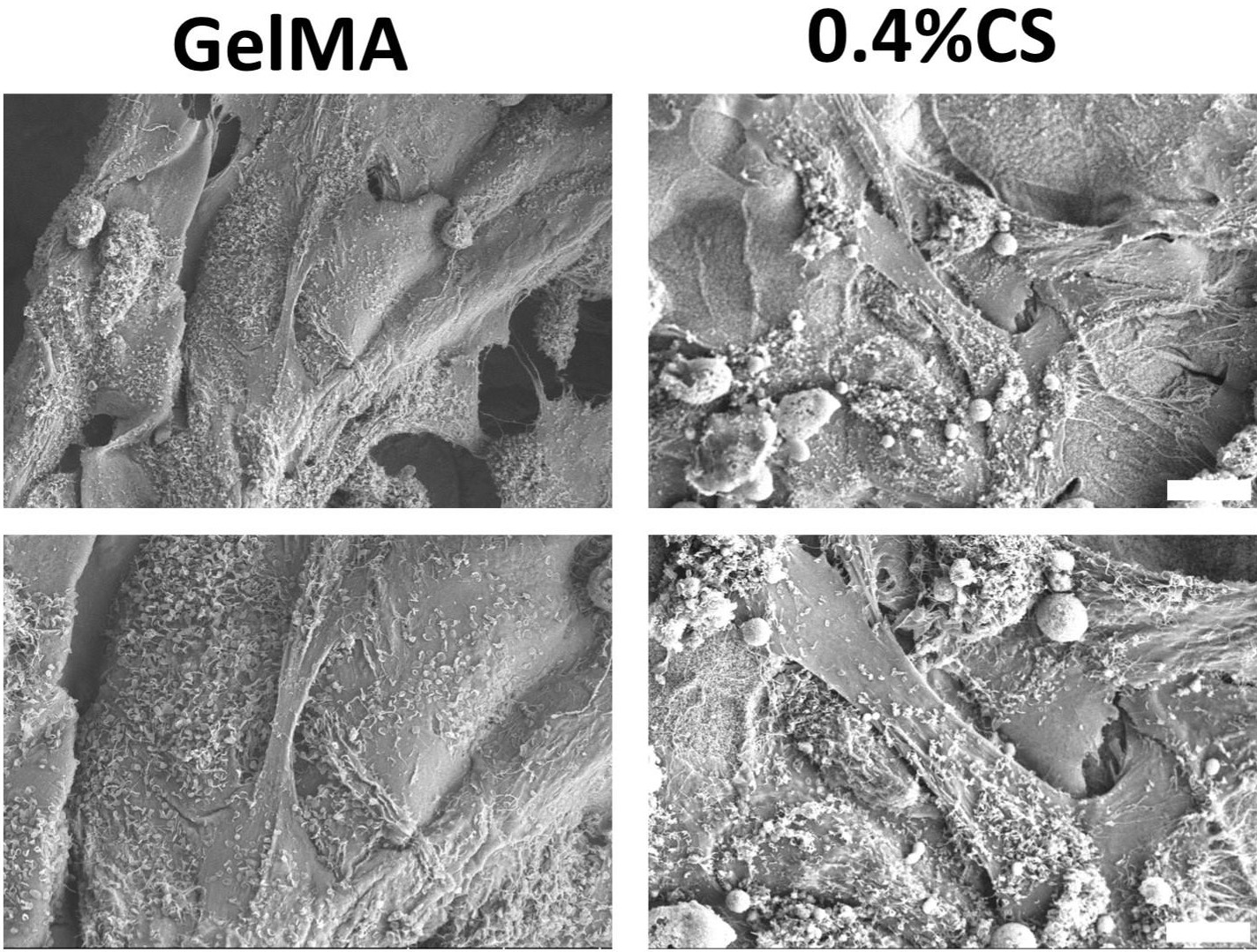
**

**Figure s3.** SEM images of HUVECs attached on GelMA and 0.4%CS scaffolds. Scale bar: 15 μm (upper row), 8 μm (lower row).


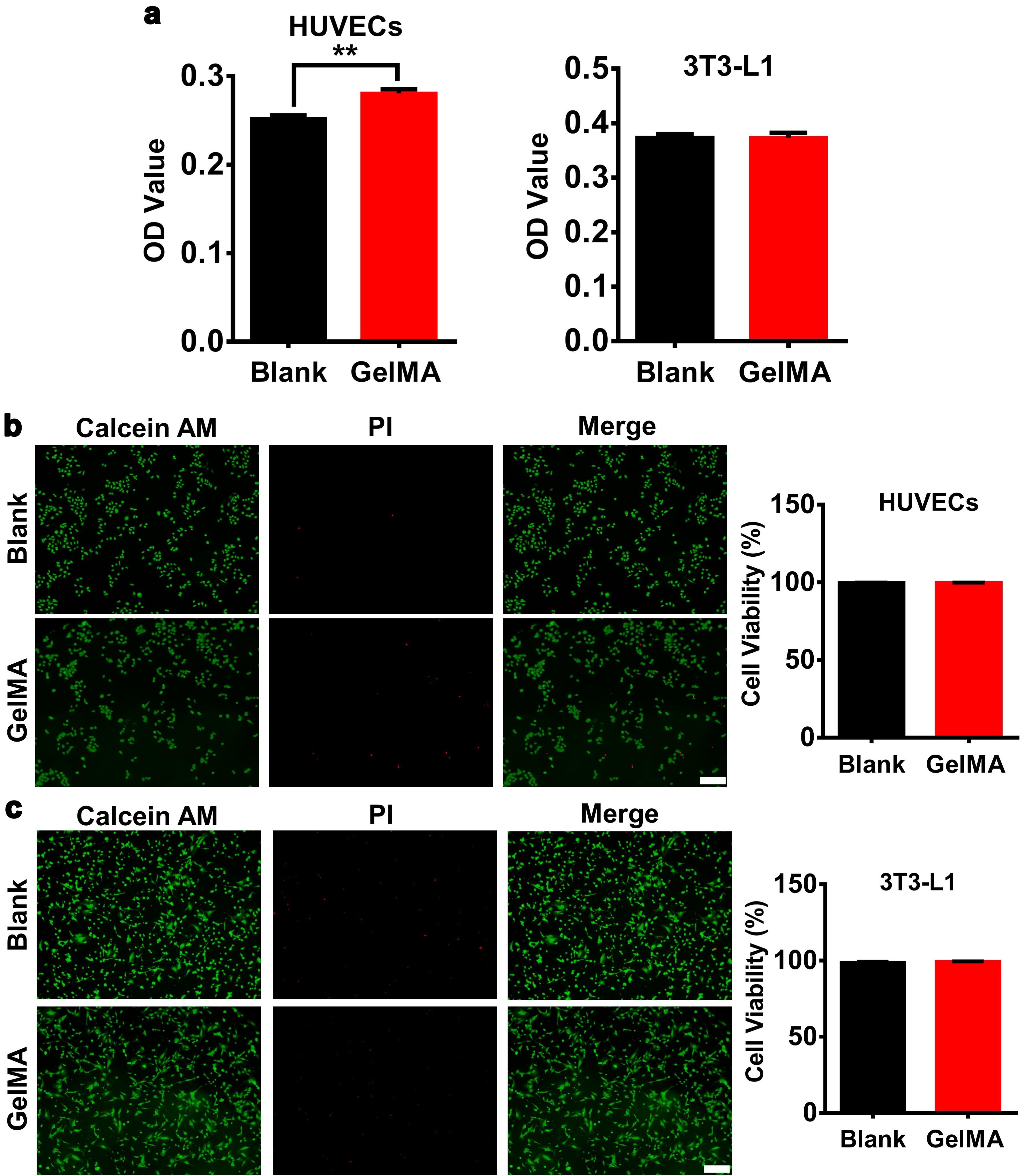


**Figure s4.** Cytocompatibility of the GelMA scaffold. (a) Cell viability of HUVECs and 3T3-L1 cells (n = 5). Fluorescent pictures of HUVECs (b) and 3T3-L1 cells (c) stained with Live/Dead assay kit (green shows the live (cells) and red shows the dead (cells). (n = 3). **P < 0.01. Scale bar: 100 μm.


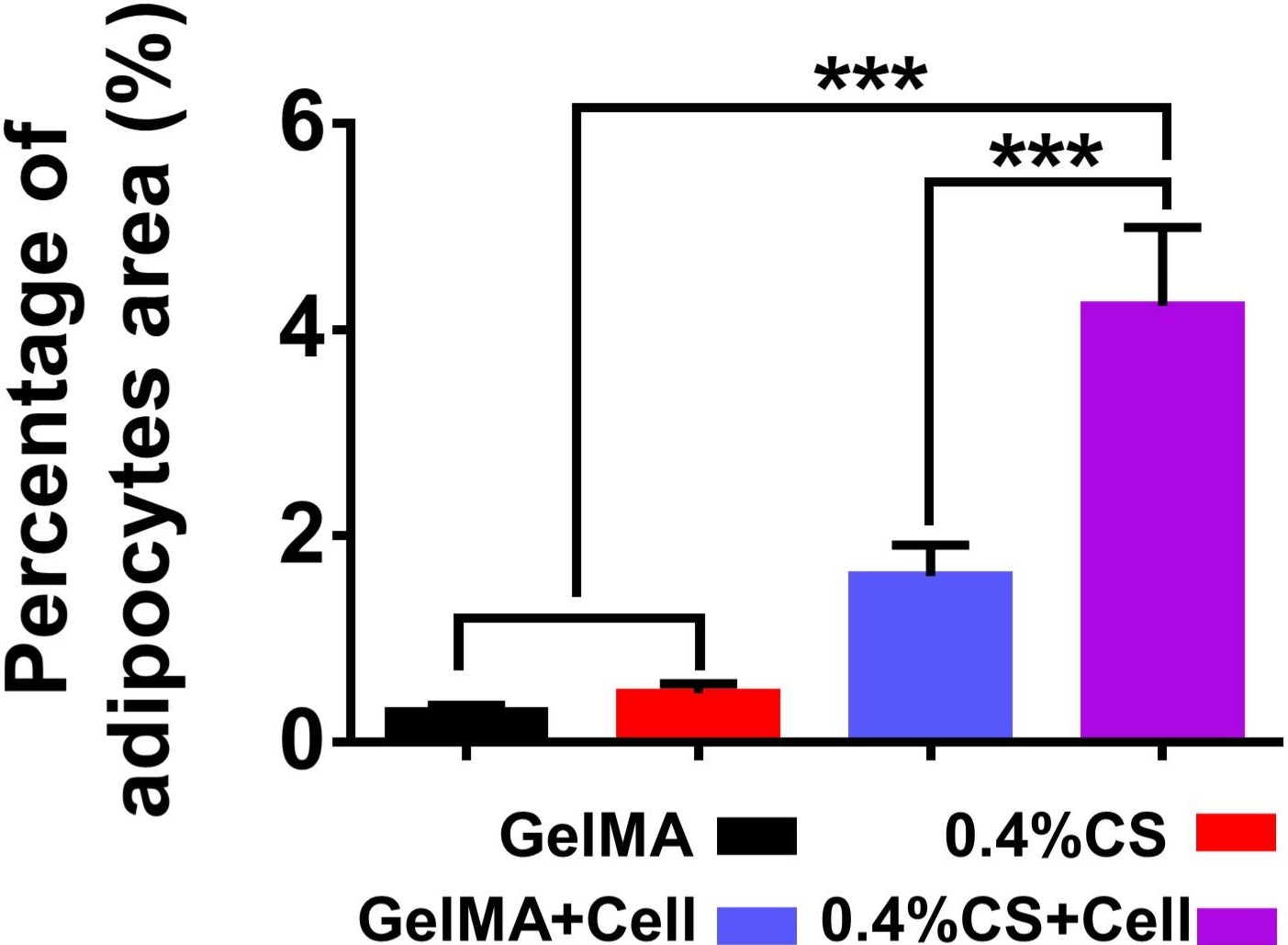


**Figure s5.** Quantitative analysis of oil red O staining in the engineered adipose tissues (n = 15, ***P < 0.001).
